# Flock composition, breeding strategies and farmers’ traits of interest evaluation of Wollo highland sheep and their F_1_ crosses

**DOI:** 10.1186/s40781-018-0173-9

**Published:** 2018-05-21

**Authors:** Tadesse Amare, Gebeyehu Goshu, Berhan Tamir

**Affiliations:** 1 Department of Animal Science, College of Agriculture, Wollo University, POBox 1145, Dessie, Ethiopia; 2 Department of Animal Production Studies and College of Veterinary Medicine and Agriculture, Addis Ababa University, POBox 34, Bishoftu, Ethiopia

**Keywords:** Flock, Breeding objective, Traits of interest, Crossbreed progenies, Wollo highland breed

## Abstract

**Background:**

Sheep production is a major component of the livestock sector in Ethiopia. The country owing to the large population of 30.70 million estimated numbers of sheep in the country and out of which about 72.14% are females, and 27.86% are males with diverse genetic resources. The real value of indigenous breeds was often under-estimated mostly due to their poor appearance and relatively low productivity. Developing countries in most cases opt for exotic breeds to increase animal productivity through crossbreeding or breed substitution without properly investigating the production potential of the indigenous breeds. The main objective of the research was to identify sheep flock composition and structure, farmers’ traits of interest and breeding objective of Wollo highland sheep, and their F_1_ crossbreed progenies.

**Results:**

Smallholder farmers’ flock synthesized from breeding ewes, breeding rams, pre-weaned ewe lambs, pre-weaned ram lambs, unproductive ewes, castrated and fattened rams, with the percentage coverage of 29.2, 13.3, 15.5, 16.5, 12.4, and 12.5%, respectively. The maximum number of flock size was 289.0 sheep per flock and higher in the third stratum. The off-take rate percentage of the three strata presented as 21.9% in 1st stratum, 12% in the 2nd stratum, and 16.4% in the 3rd stratum and higher off-take rate recorded in the first stratum. Sheep producer’s traits of interest ranked by growth rate (first), body size (second) and marketing value was third rank. Communal breeding (random mating), village based controlled breeding, mixed type and private ram controlled breeding practice were comprised of 39.7, 61.7, 52 and 71.3%, respectively. The percentages of ewes per flock composition were presented as 36.5, 27.1 and 25.5%, respectively in the 3rd stratum, 2nd stratum and 3rd stratum in the order of their importance’s.

**Conclusion:**

Genetic improvement practices at smallholder sheep producers situation was showing promising outcome with indigenous Washera F_1_ crossbred lambs and which designated for weaning rate, body size, marketing age, age at first lambing, good temperament and large litter size in the order of their rank. The contemporary breeding practice tendency indicated that, reduced flock size to improve flock productivity via crossbreeding practices.

## Background

Smallholder sheep productions are the major source of food security serving a diverse function including cash income, savings, fertilizer, socio-cultural functions, and fiber production. Sheep are particularly important for farmers in the subalpine highlands [1&18] and pastoralist/agro-pastoralist where crop production is unreliable. Moreover, its socio-cultural important sheep resources had significantly contributed for foreign currency earning and which accounting for 34% of the live animal exports [1].

Hence, sheep production is a major component of the livestock sector in Ethiopia owing to the large population of 25.4 million head [2] and the diverse genetic resources [1]. In the highlands of the country, about 75% of the sheep populations found, while the remaining 25% distributed in the lowlands [3]. Conversely, of that, Aklilu [4] reported that there is an even distribution of sheep population in the highland and lowland areas. Sheep production in the crop-livestock production systems of the highland areas has a very important role in contributing to the food security as well as generating direct cash income [1&18].

In spite of the large population of sheep and the role of sheep to both the livelihood of resource-poor farmers and the national economy at large. However, the current level of on-farm productivity in the smallholder production systems were low [5] due to various biological, environmental and socioeconomic factors involving for their poor productivity of local breeds. The reasons for productivity failures, also lack of adequate breeding practices were an important hindrance. It is necessary to identify the merit of available genetic resources, the possible integration of the animals into various production systems and to make effective use of their potential in order to quantify the existing breed differences in growth rate, growth potential, and the response of the animals to different feeding challenges. Where feed supply is a major limiting factor, it is of paramount importance to look into both biological and economical factors affecting livestock productivity [6].

The real value of indigenous breeds is often under-estimated mostly due to their poor appearance and relatively low productivity. As stated by Hodges [7], developing countries in most cases opt for exotic breeds to increase animal productivity through crossbreeding or if conditions allow by breed substitution without properly investigating the production potential of the indigenous breeds. Hence, flock composition and structure, farmer’s traits of interest, breeding objectives, and strategies not well studied under smallholder farming conditions. Hence, the present study carry out with flock composition, breeding strategies, farmer’s traits of interest and breeding objective studies of Wollo highland sheep and their crossbreeds progenies.

### The objectives of the study were

1. To analyzed existing flock size, and composition, farmer’s traits of interest at smallholder farmers breed improvement practices.
2. To assemble information on farmers’ breeding strategies, breeding ram source, and breeding objectives at smallholder farming condition.

## Methods

### Description of the study area

**Fig. 1:**
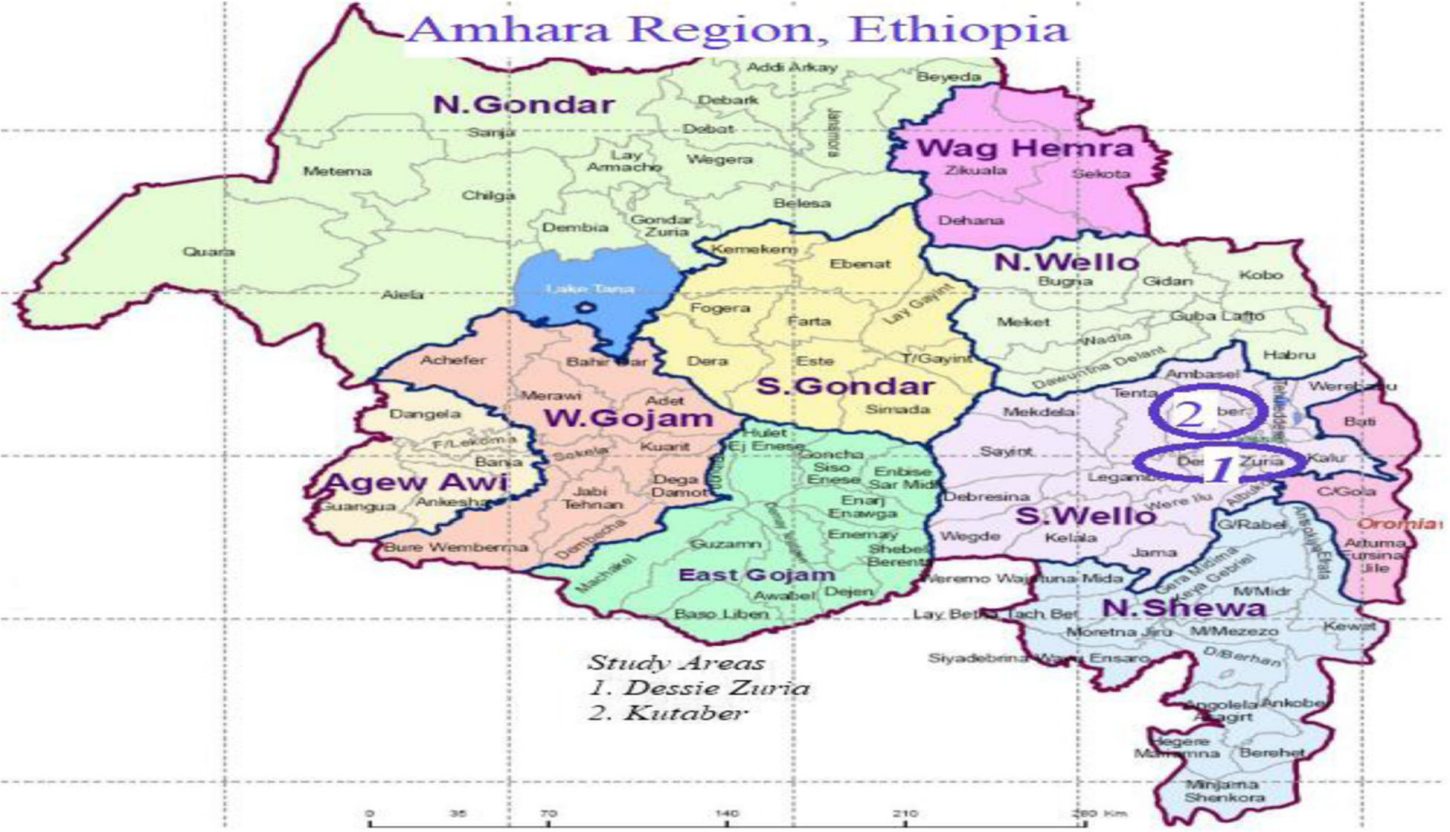
Map of the study areas in South Wollo Zone, Amhara Region, Ethiopia

The research project conducted in the two selected districts of South Wollo Zone of Amhara Region, Eastern Ethiopia. The geographical location delimited with Northern Shewa and Oromia region at the southern part, western Gojjam at western part, south Gondar at the north western part, North Wollo at the northern part, Afar Region at the northeastern part and Argobba district of Oromia Zone at the eastern part of the country (Fig. 1).

### Livestock population of the study area

Dessie Zuria and Kutaber districts had populous sheep production area from other Districts of the administrative Zone. Even though, the livelihood economy depends on crop production, much more assist by livestock production and mainly with sheep, cattle, goat, and chicken production. The districts were historically chronic food insecure area for long years ago because of shortage of rain fail and drought infected area. Sheep sold to get immediate cash income through petty trading including buying, fattening, and selling practice.

### On-farm flock management practice

**Fig. 2:**
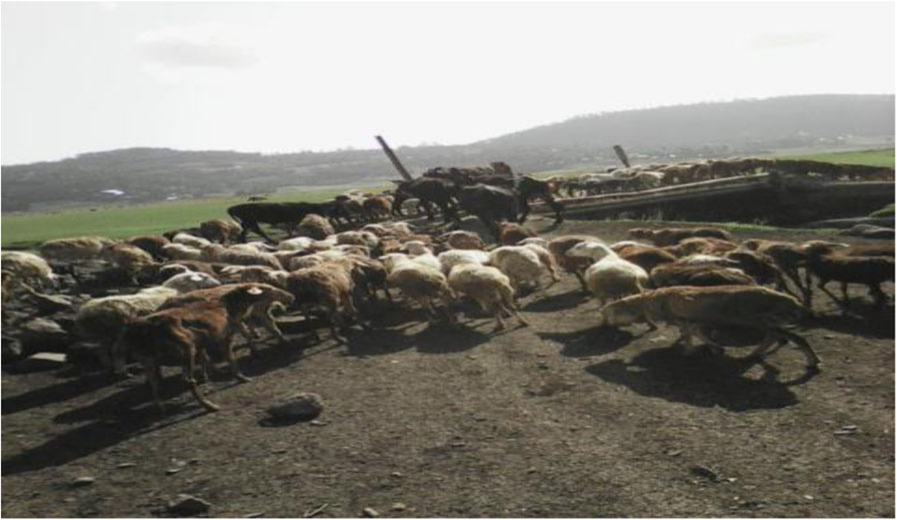
Communal mixed livestock production system in the study area

Farmers’ sheep flock management practices characterized by mixed types of crop-livestock production system. Smallholder farmers allowed their sheep flocks in a communal and private grazing land during daytime and depart during nighttime for enclosure in which they are housed together with other livestock species separated by woodlot. Some farmers who own only small flock do tie their sheep to a peg. The main feed sources for sheep were grazing on private and communal natural pasture, improved forage of oat grass (*Jerry Oats) and* vetch (*Vicia sativa*) and supplementation of non-conventional and conventional feed type. During crop harvesting times, however, sheep have access to feed crop aftermath. Some farmers give supplementation feed (wheat bran, milling and local brewery by-product, grain and grain by-product, home by-product and salt) for the pregnant and nursing ewes, suckling lambs and castrated rams. Their flock characterized by small, medium and large flock size of 1-13, 14-26 and 27 or more ewes/flock, respectively based on number of ewes per smallholder farmers’ flock (Fig. 2).

### Description of the study breeds

#### Local Wollo highland sheep breed

**Fig. 3:**
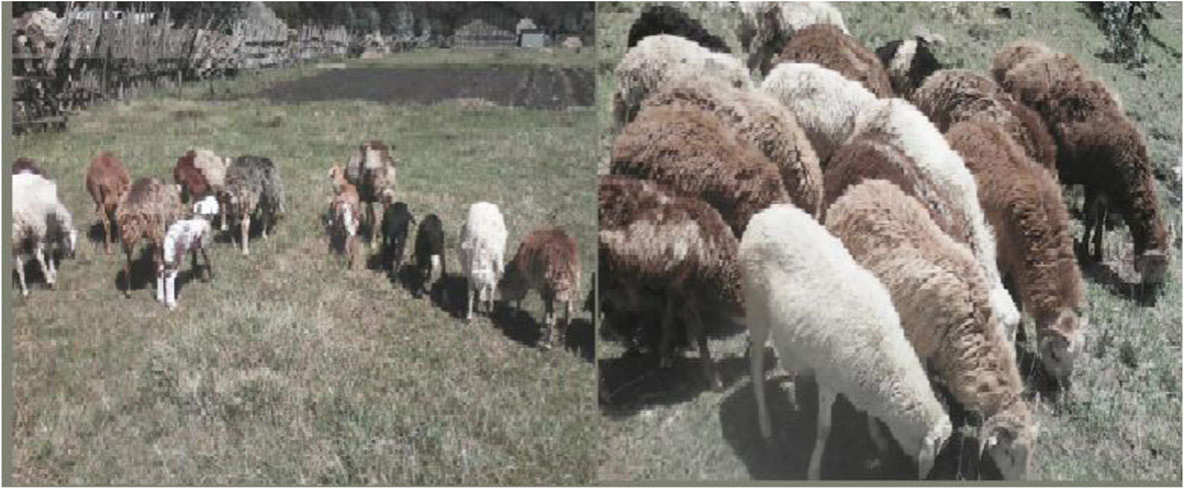
Local Wollo highland sheep breed on private natural pasture grazing area

Wollo highland sheep breed is one of the indigenous sheep breed found in the highland part of South Wollo administrative Zone at North East part of Ethiopia. They were characterized by short fat tail with short twisted/coiled end, occasionally turned up either at end, small size, well-developed wooly undercoat, predominantly black, white or brown, plain or with patches of white, black or brown, long hair with wooly undercoat and horned males [1]. It is most commonly recognized by adaptation of feed shortage, lamb survival percentage and wool production [1] (Fig. 3).

#### Washera ram crossed with Wollo highland ewes F1 crossbred progenies

**Fig. 4:**
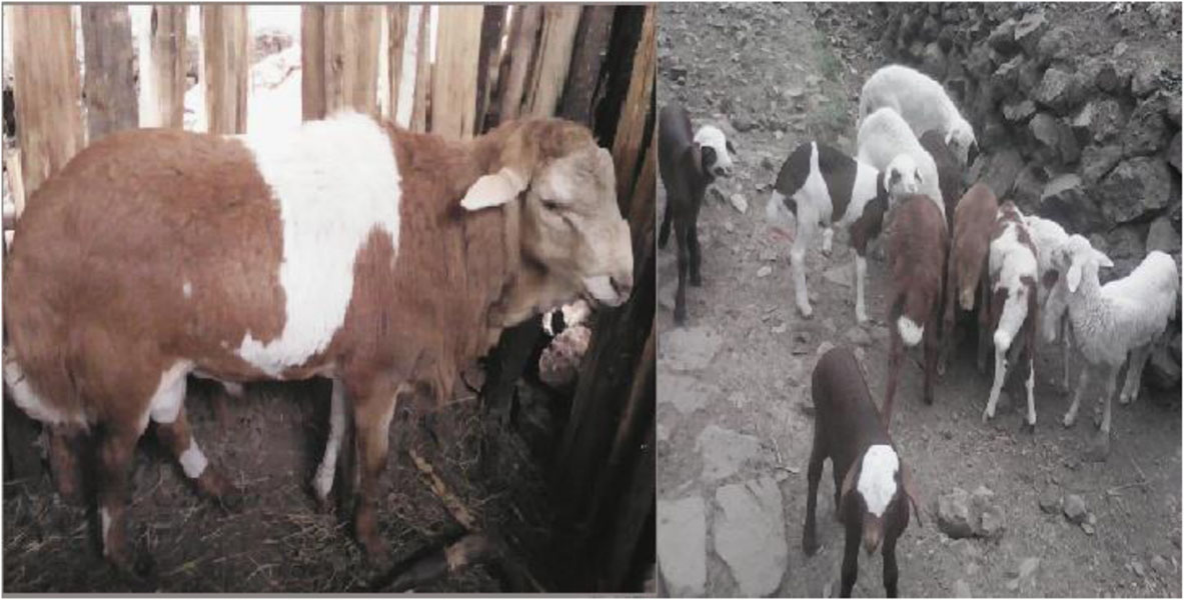
Washera ram and its crossbreds on village community based breeding scheme

Washera sheep breed is one of the indigenous sheep breeds reared by the rural farmers in the mixed crop–livestock farming systems of northwestern highlands of Ethiopia Lemma [8]. The same author reported that the Washera breed has an important genetic potential for growth and adaptation to a wide range of agro-climatic conditions. Chipman [9] has also reported their relatively fast growth rate under harsh circumstances with potentials to support smallholder farmers and national economy (Fig. 4).

#### Awassi ram crossed with local Wollo highland ewes F1 crossbred progenies

The Awassi crossbreeding projects were operates by the Debre Birhan and Amed Guya Sheep Multiplication center in Ethiopia. The multiplication center has been closed since 2004 to 2007 years because, the presence of outbreak of respiratory disease (Maedi-visna) [10] but continued later. Awassi ram and Wollo highland ewes crossbreeding practice involve importation of pure Awassi rams, for the production of 3/4 Awassi and Wollo highland crossbred progenies. Guguftu private breed multiplication center, for the distribution of 6- month-old 3/4 Awassi crossbreed ram lambs to villagers and upgrading of the village flocks to 75% Awassi [10] with back crossing were established.

### Research design

The study areas classified in to three strata. They are called as first, second and third strata and the first, second and third strata were comprised of Awassi rams F_1_crossbred progenies, Washera rams F_1_ crossbred progenies, and local Wollo highland progenies, respectively. The study areas classification performed by the assumption availability of ¾ Awassi and pure Washera rams and their F_1_ crossbred lambs, a comparable number of sheep population, agro-ecology similarity, flock management system, breeding strategies and farmers’ breeding objectives resemblance. The main feats of data collection conducted through questionnaire survey, flock monitoring, participatory focus group discussion. Thus, producers’ sheep flock size and composition, breeding objectives, sheep breeding strategies, and flock structure information gathered via questionnaire survey and group discussion.

### Sampling procedures

#### Purposive sampling method

Dessie-zuria and Kutaber Districts and six peasant association areas (four from Dessie-Zuria and two from Kutaber District) purposefully selected. The selected study areas’ were based on infrastructure accessibility, sheep population, availability of Awassi rams, Washera rams, pure Wollo highland breed and crossbreeding practices and uniformity of existing breeding strategies. After the decision of household sample size each individual sampling unites was selected by purposive sampling techniques.

#### Probability proportional to size (PPS) sampling method

Stratified probability proportional to size (PPS) sampling has the drawback of variable sample size, and different portions of the population may still be over or under-represented due to chance variation in selections. To address this problem, PPS combined with stratified approach used for representative sample size determination.

The study areas clustered into three strata. Thus, the sampling frame was drown from each stratum. Hence, the first, second and third strata consisted of 230, 370 and 675 household flocks as sampling frames, respectively. Awassi rams F_1_ crossbreed progenies, Washera rams F_1_ crossbreed progenies and local Wollo highland sheep were clustered in the first, second and third stratum, respectively. Then the sampling fractions from each stratum synthesized and sampled elements were drawn. First decided the stratum that has the smallest size then divided the size of each stratum by the smallest stratum [11 and 12] of the following equestion.

$$ \mathrm{Proportional}\kern0.17em \mathrm{size}\kern0.17em \mathrm{of}\kern0.17em \mathrm{the}\kern0.17em \mathrm{three}\kern0.17em \mathrm{strata}\kern0.17em \mathrm{were}=\frac{675}{230}:\frac{370}{230}:\frac{230}{230}\approx 3:2:1 $$

Thus, these proportions mean that; when 3 households drawn from local Wollo highland sheep breed owners, at the same time there should be 2 and 1 households draw from Washera rams F_1_ crossbred progenies and Awassi rams F_1_ crossbred progenies owners, respectively. In this way, the sample drawn as proportional to size stratified method from each stratum. According to this proportionality, the sampling ratio was calculated using addition of proportions (3 + 2 + 1), which gave as six, as value of total proportion. Finally, it divided to the smallest proportion, which equals to 1 by the total proportion of 6, and the sampling ratio were, 0.17≈0.2 $$ \left( or\raisebox{1ex}{$1$}\!\left/ \!\raisebox{-1ex}{$6$}\right.\right) $$ for all strata.

Then the elements of the sample drawn from each stratum were = 0.2*675 = 135 household flocks were selected from local Wollo highland sheep breed flocks. Whereas, 0.2*370 = 74 household flocks selected from Washera rams F_1_ crossbred progenies. Similarly, 0.2*230 = 46 household flocks selected from Awassi rams F_1_ crossbred progenies [11, 12]. Therefore, 255 sampled household flocks (135 from local Wollo highland breed, 74 from Washera rams F_1_ crossbred progenies and 46 from Awassi rams F_1_crossbred progenies) randomly selected from the three strata.

### Data collection methods

Multistage questionnaire survey used via semi-structured questionnaire and oral interview were held at each three strata household flock owners, development agents, experts, and animal health technician. The questionnaire initially pre-tested on key informants and adjusted before administrated for respondents. Stallholders sheep producer’s flock characteristics data on average flock size, number of breeding ewes, breeding rams, pre-weaning ewe lambs, post-weaning ram lambs, unproductive ewes and castrated and fattened rams, annual off take rate (slaughtered +sold and others removed from the flock by social and economical case), flock size and flock composition were gathered through questionnaire survey. While, participatory based focus group discussion data collection focused on breeding objectives, breeding strategies, farmer’s traits of interest, trait selection criterion, current breeding practice and flock structure.

On spot observation of phenotype performances data also gathered from Wollo highland sheep breed, Awassi and Washera rams F_1_ crossbred progenies to compared and contrast each other. Smallholder sheep producers were also used phenotype performances evaluation of live animal’s by body conformation, hair type, color type, drought resistance, disease resistance, marketing value, horn type, growth rate, temperament, tail type and body size were gathered through spot observation. Breeding ewe’s selection criterion of lamb survival rate, mothering ability, temperament, lamb growth rate, parental history, drought tolerance, tail type, breed type, and body conformation gathered through spot observation at smallholder sheep producer’s farm. Likewise, breeding rams selection criterions data on body size, body conformation, drought tolerance, marketing age, horn orientation, meat characteristics, growth rate, tail type, coat color, temperament, horn and hair type were also collected through on spot observation.

#### Questionnaire survey, focus group discussion and spot on observation

Single-visit, multiple-subject survey approach with semi-structured questionnaire and oral interview used for each of the three strata household flock owners, development agents and agricultural office experts. The questionnaires initially pre-tested on key informants and adjusted before administrated for the actual respondents. Smallholder sheep producers’ flock characteristics data, including flock size and composition, breeding ewes, breeding rams, pre-weaning ewe and ram lambs, unproductive ewes, castrated and fattened rams and annual off take rate (slaughtered +sold and others removed from the flock by social, cultural and economic disorder) were gathered through questionnaire survey. While, participatory based focus group discussion data collection focused on breeding objectives, breeding strategies, farmers’ traits of interest, trait selection criteria and current sheep breeding practice.

On spot observation and group discussion, data collection method also applied to phenotype characteristic of live animals’ surveillance of their body size, color type, horn type, hair type, body conformation, ear type, temperament and tail type of local Wollo highland sheep, Awassi and Washera crosses. Whereas, breeding ewes and rams selection criteria of lamb survival rate, mothering ability, temperament, lamb growth rate, parental history, drought tolerance, tail type, breed type, horn type and body conformation was gathered through spot on observation of live animals in smallholders’ flock.

### Data analysis

#### Explanatory and descriptive statistics method

Descriptive statistics of percentage, average, and graphical analytical procedures applied to compare the proportion of flock size, and composition, breeding strategies and ram breed source in the three strata. Conversely, average number of flock size, of breeding ewes, breeding rams, pre-weaning ewe lambs, unproductive ewes and castrate and fattened rams were analysed through their percentage and average values to recognize flock characteristics’ of smallholder sheep producers. Other quantitative data such as breeding strategies was analysed by relative frequency, average, and percentages of descriptive statistics procedure. Tabular and graphical representations of results also performed accordingly.

#### Ranking analysis method

Smallholder sheep producer’s breeding ewes and rams selection criteria of their breeding objectives were ranked. Phenotype traits of body conformation, hair type, color type, resistance, marketing value, horn type, growth rate, temperament, prolificacy rat, tail type, body size, age at first lambing, lambing rate and weaning rate were analyzed using index method of ranking analysis [13]. Breeding rams and ewes selection criteria based on farmer’s traits of interest were analyzed using index method of ranking analyses.

$$ Ranking\ Index=\frac{Rn\ast C1+ Rn-1\ast C2\dots +R1\ast Cn}{\sum Rn\ast C1+ Rn-1\ast C2\dots +R1\ast Cn} $$

Where, Rn = value given for the least ranked level (example if the least rank is 9th, then Rn = 9, Rn-1 = 8, R1 = 1). Cn = Counts of the least ranked level (in the above example, the count of the 9th rank = Cn, and the count of the 1st rank = C1) Musa et al. [13].

## Results

### Average flock size and composition

The percentage composition of ewes, rams, pre-weaned ewe lambs, pre-weaned ram lambs, unproductive ewes, castrated and fatten males and annual off-take rate were presented in Table 1. The overall average flock size in the given study area was 22.6 heads, however, the three stratified areas of average flock size were different. In the second stratum the average flock size was greater than the first and the 3rd strata. The average number of ewes, rams, pre-weaned ewe and ram lambs, unproductive ewes, castrated and fatten rams and annual off-take rate were also different between each strata. Except the average number of ewes and rams, the second stratum had larger than first and third stratum. The number of ewes and rams were larger in third stratum than first and second strata.

**Table 1:** Average and percentage value of flock composition at different strata

Strata	1st strata (*N* = 46)		2nd strata (*N* = 74)		3rd strata (*N* = 135)		Overall (*N* = 255)
sheep per flock	*n*	%	*n*	%	*n*	%	*n* (%)
Average flock size	19.2	100.0	26.7	100.0	21.9	100.0	22.6 (100)
Average number of ewes	5.2	27.1	6.8	25.5	8.0	36.5	6.6 (29.2)
Average number of rams	2.2	11.5	3.3	12.4	3.5	16.0	3.0 (13.3)
Pre-weaned ewe lambs	3.1	16.1	4.2	15.7	3.2	14.6	3.5 (15.5)
Pre-weaned ram lambs	3.7	19.3	4.7	17.6	2.4	11.0	3.5 (16.5)
Unproductive ewes	2.2	11.5	3.6	13.5	2.3	10.5	2.7 (12.4)
Castrated and fattened	2.8	14.6	3.9	14.6	2.5	11.4	2.7 (12.5)
Off-take rate	4.2	21.9	3.2	12.0	3.8	17.4	3.7 (16.4)

1st strata, Awassi F_1_ crosses; 2nd strata, Washera F_1_ crosses and 3rd strata, Wollo highland breed; *N*, total number of flocks; *n*, number of sheep

### Farmer’s breeding strategies and ram breed source

Village community based control sheep crossbreeding practices were being adopted approach of genetic improvement practices. This strategies, mostly applied in the second stratum than the first and third strata (Table 2). Village communities were selected their own interested ram breed type of genetic improvement of local breed through controlled communal ram use method and had their own common interest. The common interests were governed by their established rules and agreements accordingly shared breeding objective of village communities. On the other hand, private owned controlled breeding practice had the largest coverage in the third, second and first strata in the order of their importance. While, the customary of random mating breeding practice was prominent in the third stratum. However, all breeding strategies were mixed together and presented in all strata with various extent of occurrence. Hence the mixed type of breeding practices was most commonly applied in second, first and third strata in the order of their importances. Generally, private controlled, mixed type, village communities based control breeding and random mating practices presented as decreasing order of importance (Table 2).

**Table 2:** Smallholder sheep producers’ breeding strategies and rams breed source

Strata	Ram source		Village controlled breeding		Private controlled breeding		Random mating		Mixed type	
		*N*	*n*	%	*n*	%	*n*	%	*n*	%
First	Awassi crosses	212	76.0	35.8	57.0	26.9	13.0	6.1	66.0	31.1
	Own flocks (ram source)		9.0	11.9	16.0	27.6	9.0	70.2	22.0	34.0
	Project (ram source)		21.0	28.1	19.0	33.2	4.0	29.8	17.0	25.0
	Market (ram source)		35.0	45.4	22.0	39.3	–	–	22.0	32.7
	Government (ram source)		11.0	14.1	–	–	–	–	5.0	7.7
Second	Washera crosses	235	83.0	35.3	63.0	26.8	23.0	9.8	66.0	28.1
	Own flocks (ram source)		10.0	12.0	17.0	27.0	16.0	69.5	22.0	33.3
	Project (ram source)		23.0	27.7	21.0	33.3	7.0	30.4	17.0	25.8
	Market (ram source)		38.0	45.8	25.0	39.7	–	–	22.0	33.3
	Government (ram source)		12.0	14.5	–	–	–	–	5.0	7.6
Third	Wollo highland	215	26.0	12.1	94.0	43.7	83.0	38.6	12.0	5.6
	Own flocks (ram source)		3.0	11.5	26.0	27.7	58.0	69.9	4.0	33.3
	Project (ram source)		7.0	26.9	31.0	33.0	25.0	30.1	3.0	25.0
	Market (ram source)		12.0	46.2	37.0	39.4		–	4.0	33.3
	Government (ram source)		4.0	15.4	–	–		–	1.0	8.3

1st strata, Awassi crosses; 2nd strata, Washera crosses and 3rd strata, Wollo highland breed; and *N*, total number of flock owners

All breeding strategies and strata had their own source of ram breed type. Owners interested ram breed types obtained from their own flock, donor project, purchased from local markets and government supply from nucleus farm (Debre-Brehan Breed Multiplication Research Center). In the first stratum of village, communities based controlled breeding practice local market source of ram was the most common practice, followed by project donation, government supplies, and own flock ram source type accordingly their order of importance. Similarly, the first stratum of private controlled, random mating and mixed type of breeding strategies most commonly ram source was local market, own flocks and local market, respectively. While, in the second stratum of village communities based control breeding, privately owned controlled, random mating and mixed type of breeding strategies their ram source were prominently characterized by market source, own flock and both market and own flock type, respectively. On the other hand, the third stratum of village based breeding, privately controlled, random mating and mixed type of breeding strategies were most commonly applied to purchases from market, project supplied, government supplied and own flock source of rams, respectively (Table 2). Therefore, majority of rams selected from the producers’ own flock and used for random mating breeding practice. However, mixed type and private controlled breeding strategies also used rams selected from their own flocks. Generally, Private controlled breeding strategies followed by mixed type were the most common breeding strategies in the current study. The result of this study indicated that the random mating breeding strategy being reduced and replacing by controlling breeding practices.

### Breeding objectives and trait selection criterion

On-farm smallholder sheep producers comprised their own production objectives and traits of interest. In the current study first farmers were listed their interested type of traits and then each traits were ranked accordingly. Both productive and reproductive traits were the major crossbreeding goal at smallholder farming conditions. Accordingly producer’s trait selection criterion; growth rate, body size, weaning rate, body conformation, age at first lambing, lambing rate, drought resistance, color type, disease resistance, hair type**,** marketing value**,** twine rate, horn type, tail type and temperament traits were prioritized accordingly their order of importance and presented in Table 3. The most frequently used traits for reproductive and productive purpose were growth rate, body size, weaning rate, body conformation, age at first lambing and lambing rate accordingly their order of importance. Temperament, disease, and drought resistance attributes evaluated and ranked by means of the animal response towards behavioral change and incidence of disease and drought in the production condition, respectively.

**Table 3:** Indices method ranking analysis of producer’s phenotype trait of interest

Traits of interest	*N*	Index	Rank
Body conformation (large frame)	59.0	0.186	2.0
Hair type (wooly type)	28.0	0.055	9.0
Color type (without black and white)	39.0	0.060	7.0
Drought resistance (resistant)	41.0	0.068	6.0
Disease resistance (resistant)	28.0	0.055	8.0
Horn type (upward twist)	21.0	0.044	11.0
Growth rate (fast)	62.0	0.191	1.0
Prolificacy rate (twice)	26.0	0.050	10.0
Tail type (long and fat tailed)	20.0	0.040	12.0
Age at first lambing (short)	51.0	0.086	4.0
Lambing rate (fast)	42.0	0.075	5.0
Weaning rate (fast)	54.0	0.09	3.0

Growth rate, body conformation, and weaning rate traits selected by smallholder producers for sheep productivity potential improvement objectives. Therefore, farmers evaluated their live animals’ productivity on the conceptual perceptive of fast growth performance, large body fram and the rate of weaned from the dam (Table 3). Hair, color, tail, and horn type traits selected for market value addition improvement purpose based on their previous conventional marketing knowledge. Hence, wooly types of hair, color without black and white, upward twisted horn type and long fatty tailed type traits selected by smallholder farmers for marketing value advance.

Performance of drought and disease resistance considered with the potential capacity of the animals, which resist and recovered after disease and drought occurrence. Prolificacy rate, age at first lambing, lambing, and weaning rate attributes were preferred as reproductive performance improvement traits of interest. Thus, twin birth type, fast age at first lambing, short lambing interval, and fast weaning rate traits of ewes and rams were particular attributes for breeding objectives. Generally, smallholder sheep producers were select their interested traits with the impression of productivity, marketing and reproductive performance improvement practice of their animals.

## Discussion

### Average flock size

Average flock size was the major determinant factors of sheep reproductive and productive performance, income and wealth value, selection intensity, and grant for household activities. It’s a long history of traditional on-farm sheep producers were more interested in the number of animals rather than their productivity performance improvement anticipation. The overall mean number of flock size in the current study had considerable difference between the three strata (Table 1). However, the overall average flock size was slightly greater than reported by Gizaw (1) in the sub-alpine area (20.1) and lower than the same author reported in the lowland area of Ethiopia. Nevertheless, Yenesew et al. [14] reported 3.7 ± 2.4 heads of sheep per household at on-farm condition of Burie district and much lower than the current study. Tsedeke [15] was also noted that an average flock size was 7.4 in the Alaba Zone of Southern region, Ethiopia which had lower than the present study. However, according to Awigichew [16] and Samuel [17] reports, at higher altitudes of 2800-3000 m average flock size was 30 to more than 100 sheep per flocks and much higher than the current report.

Related to the present study, Gizaw et al. [18] revealed that large flock size usually had extensive sheep breeding practices and generated large number of lambs for sale. This indicated that smallholder households were usually experienced non-fattened yearling lamb sale for income generation of emergency household need, which might be negative effect on selection intensity of breed improvement practices. The large flock sizes in the subalpine sheep–barley and pastoral systems characterized by extensive mode production system, whereas in high potential cropping areas is maintenance of small flocks and production of lambs for fattening. Generally, the overall average flock size of Wollo highland sheep production had larger flock size, but becoming decreasing tendency than the previous flock size report. Therefore, the reasons might be a shortage of feed, change producers’ responsiveness from keeping large flock size to small flock size for productivity and reproductive performance improvement, grazing land limitation and increasing frequency of prevailing drought occasion. Conversely, smallholder sheep producers’ based genetic improvement practices observed and a little bit supported by government and nongovernmental organizations.

### Average flock composition

Flock structure/composition defined as the proportion (in terms of head) of the flock of sheep, which formed by different age and sex classes of animals. Flock composition in terms of age and sex classes taken as an indicator of production objectives of the owner and the production system of a given flock. Therefore, flock structure and composition was composed of the number of ewes, rams, pre-weaned ewe lambs, pre-weaned ram lambs, unproductive ewes, castrated and fattened rams in the flock (Table 1). The number of ewes, pre-weaned ewe and ram lambs, unproductive ewes, castrated and fattened male sheep, and annual off-take rate were higher in the second stratum than first and third strata. However, the number of rams was higher in third stratum than first and second strata and the reason behind this might be the number of rams not selected for breeding purposes and all ram lambs and rams presented in the flock due to the traditional way of the flock management practice. The third stratum had a high number of ewes, rams, pre-weaned ewe lambs, and unproductive ewes than first strata. However, the large number of ewes and rams in the third stratum did not show an effect on number of weaning lambs and annual off-take rate and the reason behind this might be poor fertility, and managerial condition of the flock. Whereas, the first stratum had a greater number of pre-weaned ram lambs, castrated and fattened and annual off-take rate than third stratum and it might be the better fertility performance of the breed contributed to more number of lambs weaned and which is in agreement with the idea stated by Hassen et al. [19].

In the present study, relatively larger proportion of breeding ewes per flock were observed and compared with previous result reported by Niftalem [20], Abebe [21], Tesfaye [22], Hundie and Geleta [23] and Agyemang et al. [24]. On the contrary of that the present finding had the lowest proportion of flock composition than Agyemang et al. [24] reported as 74.8% female, 22.4% entire males and 2.8% castrated for Menz sheep in the traditional sector of the Ethiopian highlands. Taye [25] revealed that flocks of Washara sheep constituted 81% females, 17.3% intact males and 1.7% castrates in traditional system in Amhara region and which had more females per flock than present result. Samuel and Belay [26] furthermore revealed that from the total sheep enumerated, 40% ewes, 7.5% rams, 51.9% lambs were comprised out of which 24.8% female and 27.11% male lambs and 0.62% castrated males which were comparatively coincide with the current finding (Table 1).

According to Taye et al. [27], the larger proportion of breeding ewes would be implied that production of a larger number of lambs, which in turn might increase the intensity of selection, and saleable lambs. The different research result indicated that the number of ewes in the flock shown greater number, but the number of lambs and ewes per flock was not proportional and which indicated that reproductive loss might be the factors to be affecting the expected number of lambs in the flock. This indicated the expected number of lambs from the given number of ewes and rams in the flock not generated. Generally, in the present study the overall flock structure and composition indicated comparatively proportional size than most other finding of in the above authors. Un proportional flock composition and structure might be indicative of external factors (feeding, disease and breeding management problems) affecting the given flock. Therefore, it might be called for researchers to search the factors affecting a given flock structure and composition.

### Breeding objectives and trait selection criterion

It the current study important attributes of producer’s breeding objectives were described and ranked by growth rate, body size, weaning rate, body conformation, age at first lambing, lambing rate, drought resistance, color type, disease resistance, hair type**,** twin rate, horn type and tail type were ranked in the order of their importance (Table 3). Kosgey [28] and Asresu et al. [29] reported that, smallholder sheep producer’s traits of interest were characterized by body size, growth performance, breed type, body conformation, temperament, color and horns were ranked in their order of importance and which is in agreement with current report of farmer’s traits of interest. Solomon et al. [30] reported that, breeding objectives of Menz, Bonga, Horro and Afar sheep production area owners were ranked by body size as first preferred traits for breeding objective achievement and in the present study growth rate was ranked as first trait of interest, so both traits are size explanatory and compatible traits. The second preference traits by the same author were growth rate in Menze, color in Bonga, tail size, and shape in Horro and Afar. However, body size in present study second ranked trait comparable with only Menz area second rank trait of Solomon et al. [30] report. Getachew [31] also reported that, about 62.5% of smallholder farmers in Menz area and 77.4% of pastoralists in Afar area were able to identify the sire of a newborn lamb by relating the lamb with the color and appearance or body conformation, respectively. Yet again, the same author stated that, lambing interval, mothering ability and milk yield in both crop-livestock and pastoral systems were important traits of interest for the choice of breeding ewes. Yadeta et al. [32] also illustrated that, farmers ranked body size, coat color and lamb survival rate as first, second and third traits of interest for selection of breeding ewes in all three agro-ecological zones. The present study was in agreement with Kosgey [28], Helen et al. [33] and Yadeta et al. [32] report of the three criteria for breeding ewes selection (Table 3).

According to Fewson [34], breeding objective is defined as “developing vital animals which will ensure that profit as high as possible under future commercial production conditions”. Solkner et al. [35], Getachew [36], and Kosgey and Okeyo [37] also stated that, subsistence farmer unlike commercial ones tend to keep animals for family need rather than purely as economic enterprise. Kosgey et al. [28] also confirmed smallholder sheep producer has kept multi-purpose animals, which produce meat, milk, wool, skin beside their ceremonial purpose and pleasure service. For that reason, it is important to consider all tangible and intangible roles of the breed, when defining breeding objectives at breed level Kosgey et al. [28].

Yadeta et al. [32] recently reported that, farmers’ trait preferences affected by agro-ecological zones. Hence, body size more preferred by highland producers, color type by midland producers and survival rate by both mid and low land sheep producers. Generally, most researchers agreed that, smallholder sheep producers were usually preferred; body size, color type, growth rate, breed type and horn type as breeding ram’s performance evaluation criterion and body size, cot color and lamb survival rate, lambing interval, twin rate and milk yield used as breeding ewes’ selection criteria. Even though, producers had multiple trait of interest, the overall interested traits were difficult to gain together from a given breed at the same period. Therefore, all interested trait have to be prioritised and considered through breeding practices and subsequently flock productivity improvement program for long-term plan of research and development activities. In general, smallholder farm producers had more focused on economical traits of interest for household income generation activities. The influence of each economical trait for genetic improvement program needed to investigate for both small scale and commercial sheep production perspectives.

### Farmer’s breeding strategies and ram breed source

Sheep breeding strategies in the current study were characterized by village community based controlled breeding, privately owned controlled breeding, random mating and mixed type of breeding strategies (Table 2). In this, finding random mating users were the lowest number of beneficiaries than the other breeding strategies. The overall percentage indicated that private controlled breeding followed by village community based controlled and mixed type of breeding practices had the largest number of beneficiaries in the order of their importance. The typical character of village community based control breeding strategy had social, cultural, common economic interest and traditionally well-defined areas in the course of breeding practice. Furthermore, breeding objectives designed and incorporated by indigenous knowledge of local communities and had developed rules and regulation of the breeding system. Private controlled breeding strategy was those sheep producers selected and controlled their own breeding rams in their flock and sometimes borrowed from their neighbor and random mating mostly controlled. Hence, each producer had their own breeding objectives and traits of interest during breeding ram and ewes selection process as parents for subsequently generation. Whereas, mixed type of breeding strategy was used all breeding strategies mixed knowingly or unknowingly. This means if a certain circumstance is accessible, the producers had their own breeding objectives, and traits of interest to be used controlled breeding strategies. However, when conditions not allowed beneficiaries might be use in random mating. Therefore, if situations are furnished those mixed type breeding strategy users had possibility to be entirely used controlled breeding strategies and so easy for developmental intervention for genetic potential improvement practices.

In agreement with the present finding, Gizaw et al. [38] revealed that village, community based control breeding strategies was applied in Menz, Bonga, Horro and Afar areas as a model for designing breeding program. However, the former genetic improvement attempts mainly focused on crossbreeding of indigenous local breeds with exotic sheep with top to down breeding approach and that not brought the expected level of result in genetic improvement practices. Both Taye [25] and Gizaw et al. [38] stated that breeding strategies were generally uncontrolled or indiscriminate mating in both Menz and Afar areas, except only to some extent in the Afar area observed in the previous progress. Thus, the major problems of conventional sheep crossbreeding approach as indicted by Workneh [39] and Markos [40] were lack of clear vision of breeding strategies, recording at smallholder level and the incompatibility of the genotype with the existing environment to bring impact. Consequently, Getachew et al. [41] reviewed that indiscriminate crossbreeding without prior analysis of crossbred suitability for a given production environment and without clear breeding objectives, presents a potential threat to better-adapted indigenous breeds. In the present study parallel with the use of exotic breeds with the supports of governmental and nongovernmental organizations; smallholder sheep producers’ trying to achieve their interested breeding objectives through introduction of new superior performance indigenous breeding rams for crossbreeding purpose from domestic market (Table 2). Selection of the superior performance, indigenous breed based on phenotype traits of ram selection criteria. Together with this random mating is trying to control by castration, fattening and selling of unwanted ram lambs before sexually maturing were common practice. The predominant practice of everywhere dissemination and selling of different breed type of breeding rams in the country to individual farmers dilutes the efforts of genetic improvement practice.

## Conclusion and recommendations

### Conclusions

The average flock size of Wollo highland smallholder’s sheep producer context had larger size but becoming decreasing than the previous report. Therefore, the reason behind this might be due to shortage of feed, producers’ awareness to change large flocks to small flock size. The other reason might be due to productivity and reproductive performance improvement strategies, grazing land limitation, and increasing frequency of prevailing drought occasion infected areas.

Smallholder sheep producers’ traits of interest represented by body size, color type, growth rate, breed type, and horn type for breeding rams’ performance evaluation criteria. On the other hand, body size, cot color and lamb survival rate, lambing interval, twin rate and milk yield traits were used breeding ewes selection criteria. The current study sheep breeding strategies characterized by village controlled breeding, private owned controlled breeding, indiscriminate mating and mixed type of breeding strategies. Currently, smallholder sheep producer’s attempt to achieve their breeding objectives through introduction of new superior performance indigenous breed rams for crossbreeding with locally available breeds.

### Recommendations

1. Smallholder farm flock productivity improvement program should be designed for long-term plan using producers’ traits of interest to bring together their production objectives.
2. All farmers’ interested traits have to be organize accordingly their significance and considered through breeding practices and need further investigation for their economical values.

## Abbreviations

CSA: Central Statistics Authority
EPA: Extension Planning Area
SPS: Sanitary and phytosanitary standards
UNCTD: United Nations Conference on Trade and Development
